# The carbapenem-resistant* Enterobacteriaceae* threat is growing: NDM-1 epidemic at a training hospital in Turkey

**DOI:** 10.1186/s12941-016-0118-4

**Published:** 2016-02-09

**Authors:** Oguz Karabay, Mustafa Altindis, Mehmet Koroglu, Onur Karatuna, Özlem Akkaya Aydemir, Ali Fuat Erdem

**Affiliations:** Department of Infectious Diseases and Clinical Microbiology, Health Sciences Institute and School of Medicine of Sakarya University, 54100 Sakarya, Turkey; Department of Medical Microbiology, School of Medicine, Sakarya University, Sakarya, Turkey; Department of Medical Microbiology, School of Medicine, Acıbadem University, Istanbul, Turkey; Department of Anesthesiology, School of Medicine, Sakarya University, Sakarya, Turkey

**Keywords:** Carbapenemase, Multidrug resistance, *Enterobacteriaceae*, Antibiotic resistance

## Abstract

**Background:**

Recently, new carbapenemases in Enterobacteriaceae strains and non-fermentative gram-negative bacilli have been reported. The New Delhi metallo‐β‐lactamase-1 (NDM-1) is a major problem around the world. The purpose of this article is to address the NDM-1 *Klebsiella pneumoniae* epidemic detected in eight cases in our hospital.

**Methods:**

Bacteria identified in this epidemic were from patients already admitted to the intensive care unit of the Sakarya University Training and Research Hospital during efforts toward establishment of infection surveillance and control program. Antimicrobial susceptibility testing of strains was performed using the VITEK 2 system (bioMérieux, France), E-test gradient strips (bioMérieux, France), and the disc diffusion test. For the metallo-beta-lactamase activity, the combined disc diffusion test and modified Hodge test as phenotypic tests were performed. To identify the resistance gene, the Xpert Carba-R kit (Cepheid Inc., USA) and an in-house multiplex polymerase chain reaction (PCR) method designed for five common carbapenemase genes (IMP, VIM, KPC, NDM-1, and OXA-48) were employed. The clonal relationship of these strains was explored by the repetitive PCR (rep-PCR, DiversiLab System, bioMérieux, France) method.

**Results:**

During the December 2014 to March 2015 period, NDM-1 positive *K. pneumoniae* strains were detected in eight patients. All of these strains were found to produce NDM-1, while two of them also revealed the presence of OXA‐48. The rep-PCR results reveal a clonal proximity of 95 % for six of the eight strains.

**Conclusions:**

Our findings suggest the tendency of NDM-1-producing strains to spread in our country as well. A carbapenem-resistant *K. pneumoniae* threat may pose a great risk to our country. It is clear that more comprehensive infection control precautions should be implemented in our hospitals.

## Background

Beta-lactamase production is one of the most common defence mechanisms of gram-negative bacteria for resistance against beta-lactam antibiotics [1]. The numbers of beta-lactamase enzymes are way more than 350. Over the years, numerous beta-lactamases and carbapenemases have been identified in gram-negative bacteria [2]. However, many centres have recently reported carbapenemase-producing gram-negative strains [3].

The treatment of carbapenemase-producing *Enterobacteriaceae* strains is very difficult. The most common mechanisms observed responsible for resistance against carbapenems in *Klebsiella* species may be listed as follows: production of particularly *Klebsiella pneumoniae* carbapenemase (KPC) in Ambler class A, metallo-beta-lactamases (MBLs) in class B (e.g. Verona integron-encoded metallo-beta-lactamase (VIM), imipenem-hydrolyzing beta-lactamase (IMI/IMP)), and OXA enzymes in Class D (beta-lactamases that hydrolize oxacillin and cloxacillin e.g., OXA-48). MBLs, can be separated into those that are chromosomally originated and those that are encoded by transferable genes [4]. All MBLs, share extra useful characters including strong carbapenemase activity, resistance to clinical beta-lactamase inhibitors (such as clavulanate), and lack of activity against monobactams [5].

Recently, production of New Delhi metallo‐β‐lactamases (NDM) have been reported in *Enterobacteriaceae*, *Acinetobacter* spp. and *Pseudomonas aeruginosa* strains. NDM-producing bacteria can spread from human to human and from water sources, foods of animal origin, and also from the polluted environment. NDM-producing strains have given rise to a number of infections resistant against almost all beta-lactamases, aminoglycosides, quinolones, nitrofurantoin, and sulphonamides. Moreover, in NDM-producing strains, such resistance can readily spread, thanks to NDM-encoding multiple chromosomal and plasmid-origin resistance genes. The carbapenemase-producing *K. pneumoniae* strain was first reported in North Carolina in 2001 [6]. Particularly over the last 5 years, the increase in the number of KPC-producing isolates is noteworthy. Despite the fact that the NDM were first identified in India in 2008, today they appear in a widespread pattern in a myriad of countries worldwide, primarily India, Pakistan, the USA, and the Balkan states. In Turkey, different centres have reported NDM-producing *K. pneumoniae* strains [7]. MBLs notifications from Turkey have increased in recent years. In a study, Karaaslan et al. studied 762 hospitalized children. Of these, 176 (23 %) were colonized with carbapenem resistant gram-negative bacilli (CR-GNB). NDM (31 %) was the second most frequent carbapenemase that was identified in *Acinetobacter baumannii* isolates. All of the 17 patients colonized with NDM-producing *A. baumannii* were newborns in the neonatal intensive care unit. Independent risk factors for CR-GNB colonization were: age <1 year, nasogastric tube placement, presence of underlying chronic diseases, ampicillin or carbapenem usage, surgical intervention [8]. In another study, Sahin and colleagues, examined 43 carbapenem-resistant strains and detected OXA-48 gene in seven isolates and NDM-1 gene in one isolate [9]. These findings indicate that carbapenem resistance due to the production of NDM-1 carbapenemase is spreading in Turkey.

The aim of this article is to report the NDM-1 producing *K. pneumoniae* epidemic involving eight patients (two of which are also harbouring the OXA-48 gene).

## Methods

### Patients and bacteria strains

Sakarya University Hospital has a 900-bed capacity and a 65-bed capacity in the intensive care unit. The Infection Control Committee (ICC) was established in our hospital in 2008. Daily regular infection surveillance studies were done by the ICC. The patients identified in this epidemic were patients already admitted to the Sakarya Training and Research Hospital intensive care unit. Eight *K*. *pneumoniae* strains were obtained from various clinical samples from the eight patients during the efforts to control the infection.

### Bacterial identification and antibiotic susceptibility testing and investigation of carbapenemase production

Identification and antimicrobial susceptibility testing of bacteria isolated from the ICU patient samples were conducted by using the VITEK 2 (bioMérieux, France) automated system and the disc diffusion test. *K. pneumoniae* strains exhibited resistance to carbapenems in VITEK 2 system. Resistance to carbapenems was also confirmed by E-test gradient strips (bioMérieux, France). Disc diffusion tests were interpreted according to the EUCAST criteria [10].

All isolates were inoculated onto ertapenem (2 mg/L) screen medium. Carbapenemase production was verified phenotypically by the modified Hodge test, and metallo-beta-lactamase activity was studied with the combined commercial disc test (Mast Diagnostics, Merseyside, UK). The modified Hodge test was performed according to the CLSI guideline using *Escherichia coli* ATCC 25922 and imipenem disk [11].

### Investigation of carbapenemase genes

**Fig. 1:**
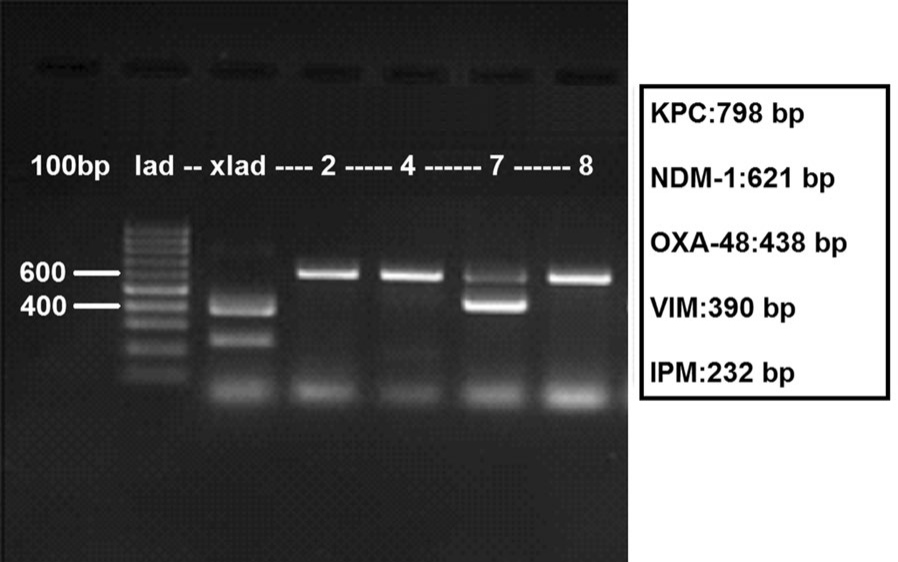
Results of multiplex PCR-gel imaging on *K. pneumoniae* strains

On the GeneXpert device, IMP1, VIM, NDM, KPC, and OXA-48 genes were studied with the Xpert Carba-R kit (Cepheid Inc., USA) as per the guidelines of the manufacturer. Identified resistance genes were verified with the multiplex PCR and gel imaging method. Additionally presence of NDM-1 genes in the strains were verified by multiplex-PCR (Fig. 1).

The five most common carbapenemase genes (*bla*_OXA-48_, *bla*_NDM-1_, *bla*_IMP_, *bla*_VIM_, and *bla*_KPC_) were investigated by an in-house multiplex PCR test. We used the primers which were previously described in the study by Poirel et al. [12]. The DNA was extracted by boiling the strains at 95 °C for 5 min. The PCR mixture was constituted of 4 μL master mix (5× HOT FIREPol Blend Master Mix Ready to Load, Solis BioDyne, Estonia), 12 μL dH_2_O, 1 μL primer mix (metabion GmBh, Germany), and 3 μL bacterial DNA (total volume 20 μL). The PCR protocol followed was 10 min at 94 °C denaturation; 35 cycles of 30 s at 94 °C, 40 s at 56 °C, and 50 s at 72 °C; and final extension for 5 min at 72 °C. PCR products were dyed with SYBR gold, loaded into 2 % agarose gel, and run on an observable real time electrophoresis (ORTE) instrument (Salubris Technica, Istanbul).

### Clonal relationship

#### Repetitive PCR

Repetitive PCR analyses were managed using the DiversiLab (DL) bacterial typing system, a semi-automated typing system based on repetitive extragenic palindromic sequence-based PCR (rep-PCR) (bioMérieux, France). The obtained DNA was quantified by spectrophotometry with the Gene Quant 1300 (GE Healthcare Europe GmbH, Milan, Italy) and diluted to 25–50 ng/ml. Diluted DNA was amplified using the *Klebsiella* fingerprinting kit (bioMérieux, France) according to the manufacturer’s instructions. Amplicons were analyzed using a 2100 Bioanalyzer (Agilent Technologies, USA). For this procedure, we used a microfluidics chip (LabChip device, Caliper Technologies Inc., Hopkinton, MA, USA) that separates DNA fragments of different sizes, resulting in chromatograms with peaks for each amplicon. Analysis was performed by Internet-based DL software 3.4, which creates virtual gel images and uses the band-based modified Kullback–Leibler distance to calculate percentage similarities. The automatically generated dendrograms, similarity matrices, electropherograms, virtual gel images, scatter plots, and selectable demographic fields were used for interpretation.

DNA band patterns from the DL system were analyzed using the DL analysis software (bioMérieux, France) with the Pearson correlation coefficient to determine the distance matrices and the unweighted pair-wise grouping with mathematical averages (UPGMA) for similarities and cluster analysis, respectively. Homologies of >97 % and 95–97 % were regarded as the parent clone (indistinguishable) and similar isolates, respectively, according to the manufacturer’s recommendations and results of longitudinal studies and comparisons [13]. A similarity value <95 % was considered to indicate a difference [14]. In addition, as in some studies, the DL system cut-off value was assumed as 90 % for *K. pneumoniae* strains for this assessment [15].

### Infection control protocols

Aggressive infection control screening protocols and laboratory testing measures were in place to identify cases. All positive patients and ICU staff members were screened for *K. pneumoniae* NDM-1 and a follow-up screening was conducted on a weekly basis. Antibiotic usage was reviewed. Positive patients were selected and isolated from negative patients. Infection control procedures in healthcare facilities, such as proper instrument and hard-surface disinfection, and a strict adherence to hand hygiene were implemented. Routine rectal swab cultures were obtained from all patients before ICU acceptance during the epidemic.

## Results

It was found that the first NDM positive (+) case was imported from a hospital located 50 km away from our hospital, with subsequent incoming patients clustering in January 2015. In this study, eight NDM-1 positive *K. pneumoniae* strains isolated from different clinical samples of eight patients were examined between December 2014 and March 2015. All of the patients were already intensive care patients of our hospital. All bacteria strains, except for a wound and a central catheter sample, were isolated from blood cultures. All of the patients with NDM-1-producing isolates in the blood culture ended up dying, except one patient in which NDM-1 (+) *K. pneumoniae* was isolated from his wound sample and who was discharged with a full recovery. The demographics and clinical facts of the patients are summarized in Table 1. Seven NDM-1 positive patients with *Klebsiella* bacteremia died (88 % mortality rate).

**Table 1:** Demographic, clinical, and laboratory data of patients

Patient	Age	Gender	Hospitalization reasons	Hospitalization start date	CR *K. pneumoniae* identification date	External centre	Isolated from	NDM/NDM + OXA	Result
1	59	F	Chronic renal failure, diabetes	10.05.2014	29.12.2014	No	Blood	NDM	Dead
2	53	F	Over carcinoma	10.05.2014	15.01.2015	No	Blood	NDM	Dead
3	67	F	Chronic renal failure, diabetes	09.09.2014	03.01.2015	Istanbul Private Hospital	Blood	NDM + OXA-48	Dead
4	74	F	Cerebrovascular disease	24.09.2014	08.01.2015	Kocaeli University Hospital	Blood	NDM	Dead
5	88	M	Hip fracture	08.10.2014	30.12.2014	Istanbul Iklim Private Hospital	Central catheter	NDM	Dead
6	28	M	Cervical fracture	15.12.2014	05.01.2015	Sakarya Adatip Hospital	Blood	NDM	Dead
7	76	F	Cerebrovascular disease, alzheimer	15.12.2014	15.01.2015	Haydarpaşa Training Hospital/Istanbul	Wound	NDM + OXA-48	Alive
8	56	F	Hypertension and fournier gangrene	16.12.2014	31.12.2014	Kocaeli University Hospital/Kocaeli	Blood	NDM	Dead

*CR* carbapenem resistant, *F* female, *M* male

We have studied the susceptibility of isolated NDM-1 (+) strains to antibiotics as potential treatment alternatives, such as amikacin, gentamicin, ciprofloxacin, colistin, and tigecycline, and found no strain susceptible to these antibiotics.

**Fig. 2:**
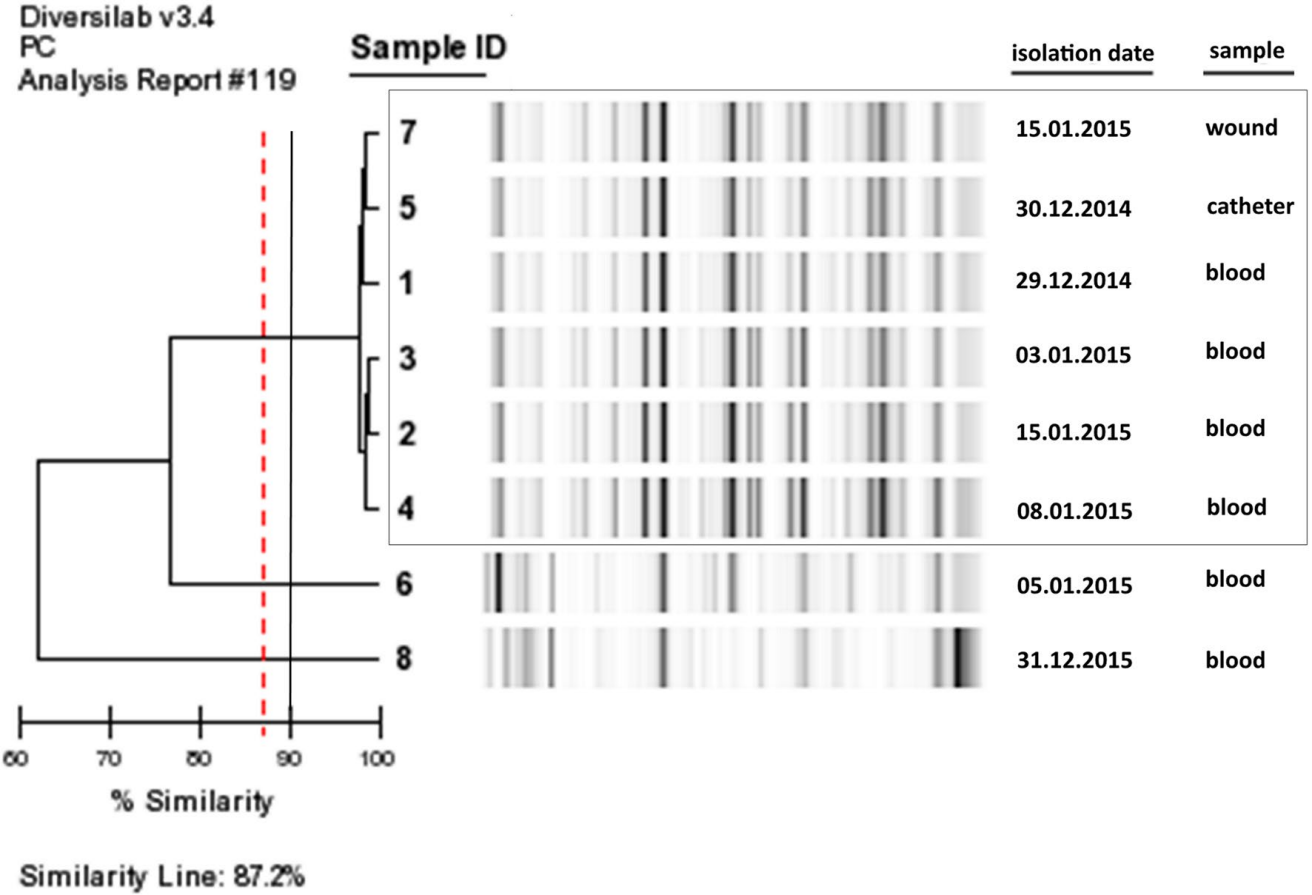
Repetitive PCR results of the *K. pneumoniae* strains

In the *K. pneumoniae* strains isolated from various clinical samples from the eight patients, the same results were obtained both with the Xpert Carba-R kit on the GeneXpert device and with the multiplex PCR gel imaging method. All NDM positive isolates were also found positive with Xpert Carba-R kit on the GeneXpert. The enzyme genes found are presented in Table 1. The rep-PCR (DL System) results reveal a clonal proximity of 95–100 % for six of the eight strains all except for the strains isolated from the samples of patients 6 and 7. In other words, the six strains are clonally indistinguishable. Since the two strains from patients 6 and 8 reveal a similarity of less than 80 %, they were found to have no clonal proximity with the others. The DL system has revealed an overall clonal similarity rate of 87.2 % for all strains (Fig. 2).

## Discussion

Some carbapenemases are reported to be more frequent in some regions. For example, while KPC-type beta-lactamases are dominant in some countries such as Greece, Israel, and USA, NDM beta-lactamases are prevalent in isolates reported from the Far East, India, and Pakistan [16]. Carbapenemase production in Turkey mostly occurs in OXA type beta-lactamases [17]. OXA-48 was reported first from Turkey, subsequently followed by reports from Middle Asia and Europe as well [18]. These isolates generally lead to multiple resistance, therefore offering very limited treatment options.

To the best of our knowledge, this study is the fourth to identify the Turkey-originating NDM existence. However, the epidemic under this study is the high scale epidemic occurring in our country with strains carrying a co-existence of OXA-48 and NDM-1. Our findings suggest the tendency of NDM-producing strains to spread in our country as well. As a matter of fact, six of the *K. pneumoniae* strains in the epidemic breaking out in our hospital were found to be clonally indistinguishable. These strains are likely to be emerging and spreading from the hospital. Therefore, the fact that such bacteria may easily spread among inpatients of the same hospital and during transfers to different medical centers should strictly be considered.

Resistance occurring with similar strains was also reported from Saudi Arabia and other Middle Eastern countries. Strains excreting NDM have the potential to rapidly spread within the country and to other countries. In a study, carbapenem non-susceptible *Enterobacteriaceae* in the Arabian Peninsula were investigated in 200 isolates collected from 16 hospitals in Saudi Arabia, Kuwait, Oman and the United Arab Emirates. In the collection, NDM-1 was the most frequently encountered carbapenemase gene (46.5 %) in their study [19]. Turkey is recently and increasingly benefiting from medical tourism. In particular, some hospitals in Istanbul admit a great number of patients, and such resistance emerging from patients admitted to the intensive care units of such hospitals is easily spreading throughout Turkey. Moreover, thousands of medical tourists come to Turkey from different parts of the world. If we cannot stop this epidemic, it will easily spread to different countries.

Bacteremia caused by such strains culminates in a high fatality rate. Given the fact that all of our cases that developed bacteraemia have died, the criticality of the threat we face can better be understood. Besides, there is unfortunately no antibiotic offering an effective treatment against infections caused by bacteria with this resistance mechanism.

It is probable that such resistance could spread to many centers throughout the country and throughout the world. It was also reported from Turkey that OXA-48 and beyond were found in *Klebsiellapneumonia*e isolates nearly one decade ago [20]. Based on the results of this study, a combination of both OXA-48 and NDM-1 type resistance could easily spread. This situation can cause significant morbidity. Therefore, the presence of NDM and OXA-48 in carbapenem-resistant *K. pneumoniae* strains isolated from patients in the intensive care units in Turkey should be explored. Moreover, our hospitals should create awareness about this type of resistance, and measures to prevent the resistance from spreading should be done immediately. A forceful infection control and prevention strategy should be implemented, and should contain the reinforcement of hand hygiene, contact precautions, and initial recognition of CRE through the use of direct surveillance. We could not sequence typing of NDM producing *K. pneumoniae* due to project budget.

## Conclusions

The bacteremia mediated by NDM-1-producing *K. pneumoniae* was observed for the first time in India in 2008, and strains with the same resistance mechanism could spread up to Turkey and may likely spread to many other countries therefrom. Moreover, we found that the NDM-1 resistance of *K. pneumoniae* isolates also can be accompanied by OXA-48. For this agent with limited treatment options, we should comply with measures for controlling infection. We believe that the treatment of infectious diseases in the intensive care units of our country could be much harder in the coming years.
